# Practical Guidelines for Preventing Congenital Syphilis

**DOI:** 10.1155/S1064744993000262

**Published:** 1993


Infectious Diseases in Obstetrics and Gynecology 1:115–117 (1993)

© 1993 Wiley-Liss, Inc.

### Practical Guidelines for Preventing Congenital Syphilis

A recent case of congenital syphilis at our hospital made us aware of the many potential pitfalls in the prevention of this disease. The patient in question presented in labor at term, and the intern noted a negative VDRL at the first prenatal visit. The attending physician on call (one of the authors) noted the low birth weight; however, the newborn had good Apgar scores and a normal cord pH. Another resident rotating through our department did not check the VDRL drawn post partum before discharging the mother. The laboratory did not perform the VDRL until 3 days after the patient delivered because it was a long holiday weekend. When the laboratory notified the floor, the nursing staff said, "Don't worry about it" because the patient had already been discharged. The case was finally tracked down through our local public health department, and the mother had the classic palmar-plantar rash of secondary syphilis. Therefore, the neonate was readmitted for treatment of congenital syphilis.

This editorial only emphasizes what many readers already know—cases of early syphilis among reproductive-age women have risen rapidly in the past few years, and subsequently the number of cases of congenital syphilis has reached record numbers (Fig. 1). Some of the news is good, with overall cases of syphilis declining over 1990 to 1992. In our area, the number of cases has remained high, with Kansas City being 6th and St. Louis 1st in per capita syphilis cases in 1992 (Table 1). The current epidemic has been characterized as urban, heterosexual, and related to illicit drug use.[1] There is evidence that syphilis has diffused into rural areas as well. The Centers for Disease Control (CDC) reported six cases in a rural western Kansas county in 1992, the first cases reported since 1989.[2] Two of the these cases were pregnant women.

Unfortunately, physician error can play a role in cases of congenital syphilis. In a recent case series from Detroit,[1] errors in detection, treatment, or diagnosis were responsible for many of the 51 cases of congenital syphilis. In 18 cases, no serological test for syphilis was done on maternal or cord blood. In two-thirds of cases with positive serological tests, no treatment was initiated primarily because physicians were not aware of the positive results (similar to our case). Three cases were due to inadequate therapy.

We wish to present these pragmatic suggestions for the prevention of congenital syphilis:

1. *Adequate screening*. The recently published 19th edition of *Williams' Obstetrics* states that a nontreponemal test for syphilis should be done at the first prenatal visit and at delivery. The authors also state that in half of all cases of congenital syphilis the mothers receive adequate prenatal care but are not screened according to the above suggestions. In areas of high prevalence, an additional screen should be done early in the 3rd trimester. At our institution, we obtain a VDRL at the time of the glucose challenge, thereby detecting and treating several cases of syphilis.
2. *Laboratory measures*. A nontreponemal test for syphilis should be done in a timely manner (5 or 6 times a week regardless of holidays) on each maternal

Editorial

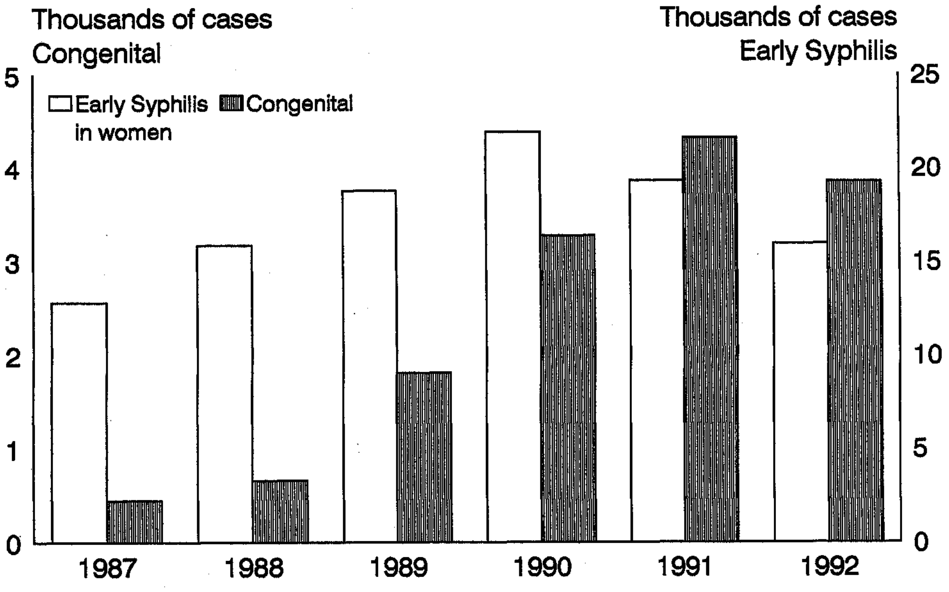

Fig. 1. Congenital syphilis, 1987–1992. Source: CDC.

TABLE 1. Early syphilis in selected cities in the United States, 1992

| City | Rank | Cases/100,000 population |
|---|---|---|
| St. Louis, MO | 1st | 153.3 |
| Kansas City, MO | 6th | 64.8 |
| Chicago, IL | 7th | 64.1 |
| Detroit, MI | 14th | 51.1 |
| Miami, FL | 31st | 16.8 |

Source: CDC

sample. Maternal samples should be given priority. Laboratory personnel should regard a reactive serological test as a "panic" value and contact the responsible physician immediately.

3. *Penicillin*. Penicillin is the treatment of choice for syphilis in pregnancy. Erythromycin does not cross the placenta and thus does not prevent congenital syphilis. Penicillin-allergic patients should undergo inpatient penicillin desensitization.

4. *Education*. All health care providers including nurses, residents, and community physicians must be made aware that syphilis is on the rise. No mother should be released from the postpartum unit without a serological test result. As a result of the above case, we have devised a "worksheet" for the postpartum patient that must be completed prior to discharge. This worksheet contains maternal blood type, hemoglobin, rubella status, plans for contraception, and results of hepatitis-B surface antigen test.

Congenital syphilis is a preventable disease. Errors in patient care are a factor on the current epidemic, and implementing some form of the above guidelines along with increased awareness should help decrease the incidence of congenital syphilis.

### REFERENCES

1. Berry M, Dajani A: Resurgence of congenital syphilis. Infect Dis Clin N Am 6:19–29, 1992.
2. CDC: Syphilis—Ford County, Kansas, 1992. MMWR 41:644–645, 1992.

*Kevin A. Ault and Sebastian Faro*
